# Reducing the Sodium Intake of Patients With Chronic Kidney Disease Through Education and Estimating Salt Excretion: A Propensity Score Matching Analysis

**DOI:** 10.7759/cureus.43510

**Published:** 2023-08-15

**Authors:** Hisato Shima, Takuya Okamoto, Manabu Tashiro, Tomoko Inoue, Seiichiro Wariishi, Kazuyoshi Okada, Toshio Doi, Takeshi Nishiuchi, Jun Minakuchi

**Affiliations:** 1 Kidney Disease, Kawashima Hospital, Tokushima, JPN; 2 Laboratory, Kawashima Hospital, Tokushima, JPN; 3 Cardiovascular Surgery, Kawashima Hospital, Tokushima, JPN; 4 Cardiovascular Medicine, Kawashima Hospital, Tokushima, JPN

**Keywords:** propensity score matching, education, chronic kidney disease, hypertension, salt intake

## Abstract

Background: Japanese people traditionally consume high quantities of salt. This study aimed to investigate the effects of educating patients with chronic kidney disease (CKD) on simple methods for reducing their daily dietary salt intake.

Methods: This single-center, retrospective observational study included 115 outpatients with CKD at Kawashima Hospital (Tokushima, Japan). One physician routinely recommended that patients should reduce their salt intake and provided tips for salt restriction. The physician estimated the patients’ daily salt intake using spot urine samples at each medical examination (education group; n = 61). The other physicians’ outpatients only received dietary guidance on recommended salt intake (control group; n = 54). The estimated 24-hour urinary sodium excretion (24hUNaV) and 24-hour potassium excretion (24hUKV) were calculated using Tanaka’s equation.

Results: Estimated 24hUNaV was positively correlated with body mass index (BMI), estimated 24hUKV, and urinary Na/K ratio. The patients in the education group were younger and had a lower BMI, higher estimated glomerular filtration rate, and lower systolic blood pressure (SBP). Using 38 pairs of patients obtained by propensity score matching with these variables, estimated 24hUNaV, estimated 24hUKV, and diastolic blood pressure (DBP) after one year were significantly reduced in the education group.

Conclusion: A simple salt reduction education may reduce salt intake in outpatients with CKD.

## Introduction

Dietary salt intake is closely associated with hypertension [1]. Excess sodium intake increases the risk of high blood pressure (BP) and cardiovascular events [1,2]. Japanese people traditionally consume high quantities of salt [3], with the average salt intake being 10.9 g/day for men and 9.3 g/day for women, as per latest survey results [4]. The hypertension guidelines set by the Japanese Society of Hypertension (JSH) in 2019 proposed a salt intake goal of <6 g/day [5]. Therefore, reductions in dietary salt intake are urgently needed. In patients with chronic kidney disease (CKD), high BP is a common condition attributed to sodium sensitivity [6]. Several interventions have been reported to be effective in spreading awareness regarding the benefits of reducing dietary salt intake; however, the reproducibility and feasibility of these interventions remain unclear [7]. One of the factors that affect the achievement of reducing dietary salt intake in patients with hypertension is the lack of opportunity to understand the amount of their salt intake. Generally, salt intake is not routinely measured during a medical examination. Although nutritional guidance by a dietician is important, some patients do not wish to receive dietary counseling owing to time constraints. Hence, this study aimed to investigate the effects of educating patients with CKD on simple methods for lowering their daily sodium intake on salt intake and BP.

## Materials and methods

Study design

This single-center, retrospective observational study included 115 outpatients with CKD at Kawashima Hospital (Tokushima, Japan). One physician routinely recommended that patients reduce their salt intake and provided tips for salt restriction (Table 1).

**Table 1:** Tips for salt restriction

No.	Tip
1	Get used to the bland taste.
2	Be careful about the quantity of pickles and soups consumed.
3	Use salt effectively.
4	Eat food with seasonings incorporated instead of adding seasonings.
5	Use souring agents.
6	Make the food spicy.
7	Use seasoning ingredients.
8	Use fragrant flavoring agents.
9	Use oil.
10	Be careful of nibbles for drinks.
11	Be careful of paste products and processed foods.
12	Try not to eat too much.

The physician estimated the patients’ daily salt intake using spot urine samples at each medical examination. These patients comprised the simple salt-reduction education group (education group) in this study. The other physicians’ outpatients with CKD only received dietary guidance on recommended salt intake, but they did not receive tips for salt restriction or have their daily salt intake estimated (control group). In the control group, nutritional guidance was provided not by dietitians but physicians and did not include the content of tips for salt restriction. In this study, spot urine samples, BP, and body mass index (BMI) data were collected at baseline and one year after the start of salt reduction education. We excluded patients who used steroids and those who started medications that may affect sodium reabsorption, such as loop or thiazide-type diuretics, within one week of urinalysis. The estimated 24-hour urinary sodium excretion (24hUNaV) and 24-hour potassium excretion (24hUKV) were calculated using Tanaka’s equation [8]. The office BP was measured in accordance with the JSH guidelines [5]. 

This study was approved by the Ethics Committee of Kawashima Hospital (approval no. 0871). All clinical investigations were conducted in accordance with the principles of the Declaration of Helsinki and Japanese ethical guidelines.

Statistical analyses

All statistical analyses were performed using JMP version 16 (SAS Institute Inc., Cary, NC, USA). Data are presented as medians with interquartile ranges for continuous variables and numbers (percentages) for categorical variables. Continuous variables were compared using the Student’s t-test or Mann-Whitney U test, as appropriate. Categorical variables were evaluated using the chi-squared test or Fisher’s exact test as appropriate. Spearman correlation tests were used for correlation analyses. Propensity score matching was performed to minimize bias due to confounding variables. The propensity score calculated using multivariate logistic analysis included the following variables: age, BMI, estimated glomerular filtration rate (eGFR), and systolic BP (SBP). Matching was performed using a 1:1 ratio with 0.20-width calipers. A p-value < 0.05 was considered statistically significant.

## Results

**Figure 1:**
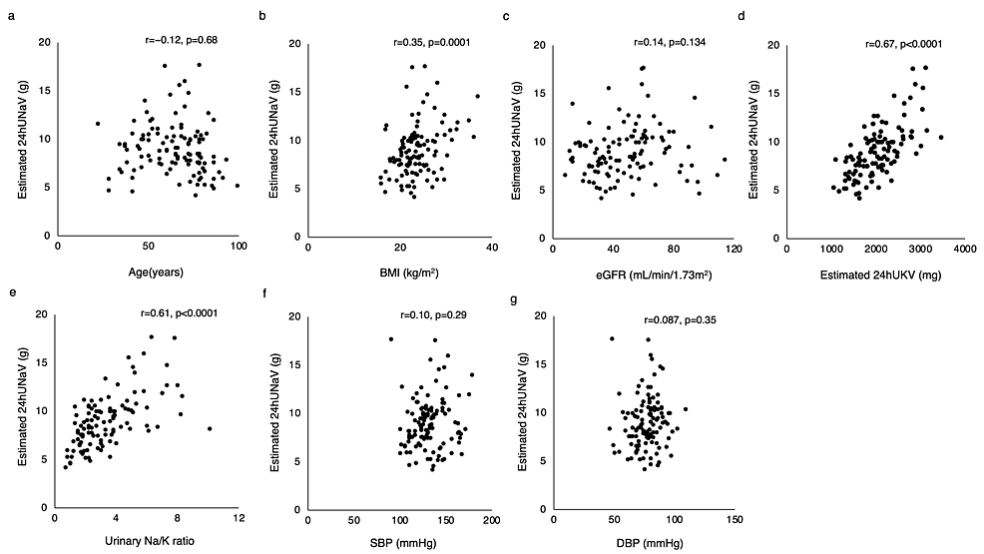
Correlation between the estimated 24hUNaV and (a) age, (b) BMI, (c) eGFR, (d) estimated 24hUKV, (e) urinary Na/K ratio, (f) SBP, and (g) DBP 24hUNaV: 24-hour urinary sodium excretion, 24hUKV: 24-hour potassium excretion, BMI: body mass index, SBP: systolic blood pressure, DBP: diastolic blood pressure, eGFR: estimated glomerular filtration rate

We analyzed the correlation between the estimated 24hUNaV and the variables. In the Spearman correlation analysis, the estimated 24hUNaV was positively correlated with the BMI (r = 0.35, p = 0.0001), estimated 24hUKV (r = 0.67, p < 0.0001), and the Na/K ratio (r = 0.61, p < 0.0001) (Fig. 1b, 1d, 1e, respectively). There were no correlations with age, eGFR, SBP, or diastolic BP (DBP) (Fig. 1a, 1c, 1f, 1g, respectively). 

**Figure 2:**
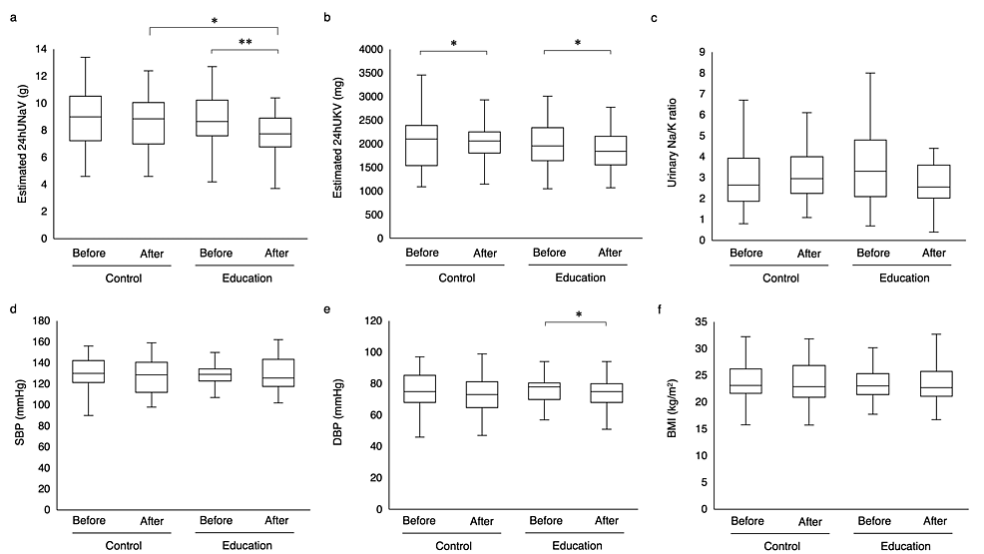
After propensity score matching with age, BMI, eGFR, and SBP. Box-and-whisker plot of the (a) estimated 24hUNaV, (b) estimated 24hUKV, (c) urinary Na/K ratio, (d) SBP, (e) DBP, and (f) BMI of the two groups before and after education 24hUNaV: 24-hour urinary sodium excretion, 24hUKV: 24-hour potassium excretion, SBP: systolic blood pressure, DBP: diastolic blood pressure, BMI: body mass index, eGFR: estimated glomerular filtration rate *p < 0.05, **p < 0.01 versus before or control

A comparison of the baseline demographic data and clinical characteristics between the education and control groups is presented in Table 2. Compared with the control group, the patients in the education group were younger (63.0 years (46.5-73.5 years) versus 72.5 years (55.8-78.0 years); p < 0.001), had a lower BMI (22.6 kg/m^2^ (20.3-24.3 kg/m^2^) versus 23.8 kg/m^2^ (22.1-26.3 kg/m^2^); p = 0.042), higher eGFR (53.0mL/min/1.73 m^2^ (32.5-69.0 mL/min/1.73 m^2^) versus 42.0 mL/min/1.73 m^2^ (29.8-55.5 mL/min/1.73 m^2^); p = 0.032), and lower SBP (127.0 mmHg (115.0-133.0 mmHg) versus 136.5 mmHg (123.0-149.8 mmHg); p < 0.001). No significant differences were found among the remaining characteristics. A total of 38 pairs of patients were obtained by propensity score matching with age, BMI, eGFR, and SBP. After matching, there were no significant differences in baseline characteristics between the two groups (Table 2). The estimated 24hUNaV was significantly reduced in the education group (Fig. 2a). One year later, it was lower in the education group than in the control group (Fig. 2a). The estimated 24hUKV was significantly reduced in both groups (Fig. 2b). DBP was significantly reduced in the education group (Fig. 2e). The urinary Na/K ratio, SBP, and BMI did not significantly change in either group (Fig. 2c, 2d, 2f, respectively). 

**Table 2:** Baseline characteristics of the patients before and after propensity score matching BMI: body mass index, eGFR: estimated glomerular filtration rate, 24hUNaV: 24-hour urinary sodium excretion, 24hUKV: 24-hour urinary potassium excretion, SBP: systolic blood pressure, DBP: diastolic blood pressure, ARB: angiotensin II receptor blocker, ACEI: angiotensin-converting enzyme inhibitor *p < 0.05 when comparing the education group versus control group

Characteristics	Before matching				After matching		
	Control group	Education group	p		Control group	Education group	p
n	54	61			38	38	
Age (years)	72.5 (55.8-78.0)	63.0 (46.5-73.5)	<0.001^*^		69.0 (51.5-78.0)	65.5 (57.8-77.0)	0.97
Male sex (%)	33 (61.1)	32 (52.5)	0.35		25 (65.8)	22 (57.9)	0.48
BMI (kg/m^2^)	23.8 (22.1-26.3)	22.6 (20.3-24.3)	0.042^*^		23.1 (21.4-25.5)	22.9 (21.1-24.5)	0.62
eGFR (mL/min/1.73 m^2^)	42.0 (29.8-55.5)	53.0 (32.5-69.0)	0.032^*^		45.5 (30.8-55.5)	48.5 (24.8-62.3)	0.63
Sodium (mmol/L)	141 (140-142)	140 (139-142)	0.053		141 (140-142)	140 (139-142)	0.071
Potassium (mmol/L)	4.4 (4.1-4.6)	4.2 (4.0-4.7)	0.38		4.5 (4.1-4.6)	4.3 (4.1-4.8)	1.00
Estimated 24hUNaV (g)	9.2 (7.3-10.6)	8.5 (7.3-10.2)	0.36		9.6 (7.8-10.6)	8.8 (7.6-10.1)	0.36
Estimated 24hUKV (mg)	2063 (1607-2390)	1959 (1636-2268)	0.47		2125 (1796-2456)	1962 (1644-2286)	0.26
Urinary Na/K ratio	3.0 (2.1-4.7)	2.8 (1.9-4.4)	0.67		3.0 (2.1-4.1)	3.1 (2.2-4.7)	0.81
SBP (mmHg)	136.5 (123.0-149.8)	127.0 (115.0-133.0)	<0.001^*^		129.5 (118.8-139.8)	128.0 (123.0-134.0]	0.89
DBP (mmHg)	78.5 (68.0-86.0)	78.0 (70.0-82.0)	0.86		76.0 (68.0-85.3)	79.0 (71.5-85.3)	0.37
Antihypertensive drugs (%)	41 (75.9)	41 (67.2)	0.30		32 (84.2)	29 (76.3)	0.57
ARB or ACEI (%)	38 (70.4)	32 (52.5)	0.058		29 (76.3)	22 (57.9)	0.14
Calcium channel blockers (%)	22 (40.7)	29 (47.5)	0.46		17 (44.7)	23 (60.5)	0.17
Diuretics (%)	9 (16.7)	9 (14.8)	0.80		6 (15.8)	3 (7.9)	0.48
α blockers or β blockers (%)	6 (11.1)	8 (13.1)	0.78		4 (10.5)	7 (18.4)	0.52
Mineralocorticoid receptor antagonist (%)	0 (0)	1 (1.6)	1.00		0 (0)	0 (0)	1.00
Antidiabetic drugs (%)	7 (13.0)	13 (21.3)	0.33		5 (13.2)	9 (23.7)	0.38
Lipid-lowering drugs (%)	28 (51.9)	28 (45.9)	0.52		22 (57.9)	18 (47.4)	0.36
Hyperkalemia improving agents (%)	5 (9.3)	2 (3.3)	0.25		3 (7.9)	2 (5.3)	1.00

 

There were no significant differences in the proportion of the estimated 24hUNaV < 6 g/day between the two groups before and after dietary guidance on salt (Table 3). 

**Table 3:** Differences in the estimated 24-hour urinary sodium excretion < 6 g/day in each group before and after salt reduction education

	All	Control group	Education group	p
n	76	38	38	
Before education (%)	7 (9.2)	3 (7.9)	4 (10.5)	1.00
After education (%)	7 (9.2)	3 (7.9)	4 (10.5)	1.00

## Discussion

In this study, we found that a simple salt reduction education is a novel approach of reducing sodium intake in patients with CKD. This approach is available for self-monitoring of salt intake and increasing awareness at each medical examination regarding salt reduction. Salt intake in Japan is much higher than that in other countries because of the habitual consumption of traditional high-salt Japanese foods [9]. A previous report suggested that awareness of the importance of salt restriction is not associated with actually doing so in outpatients [10]. Long-term compliance with salt restriction has also been reported to be poor in Japanese patients with hypertension [11]. A simple salt reduction education includes taking measurements of urinary salt excretion at each medical examination, which may encourage salt restriction.

Salt sensitivity is defined as BP susceptibility to dietary salt intake where changes in BP parallel to changes in salt intake [12]. There is currently no standardized method for diagnosing salt sensitivity. There are large variations in the salt sensitivity of BP among patients with hypertension [13]. A meta-analysis showed that a salt reduction of 1 g/day lowered SBP by 1 mmHg in patients with hypertension [14]. In our study, sodium intake significantly decreased from the baseline, whereas SBP did not change in the education group. There are two possible reasons for this: (1) The BP in this study was within the normal range, and the estimated 24hUNaV reduction was 0.7 g/day, which may have been too small of a change to affect BP. (2) This may be due to a significant decrease in the estimated 24hUKV. Higher potassium intake has been shown to mitigate BP response to higher sodium intake [15]. In the case of hyperkalemia, restricting dietary potassium intake is often recommended for patients with CKD [16]. In such cases, dietary guidance may include potassium restrictions. This may be associated with a paradoxical reduction in urinary potassium in individuals who have adhered to a low-sodium diet in this study. A higher dietary Na/K ratio is associated with a higher risk of cardiovascular mortality [17]. Our study did not reveal any changes in the Na/K ratio with the salt reduction education level. Because aldosterone effects could influence the urinary Na/K ratio in subjects with hypertension, we investigated the potassium level, mineralocorticoid receptor blockers' use rate, and hyperkalemia-improving drugs' use rate. However, these factors did not significantly change in either groups. Further studies are needed in a large group with CKD and hypertension to investigate the effect of a simple salt reduction education on BP.

In this study, we showed that salt intake was closely related to BMI, as previously reported [18]. Several factors contribute to salt sensitivity, including genes, ethnicity, age, sex, BMI, and renal function [13,19,20]. After matching these factors, including age, BMI, and eGFR in this study, there seemed to be little difference in salt sensitivity between the two groups. Our study also revealed that the achievement rate of the target salt intake (<6 g/day) was very low after salt reduction education. It appears to be very difficult to attain this target salt intake [2]. It is possible that associated medical conditions may also aggravate the impact of daily salt intake, such as hypertension, heart failure, coronary artery disease, or other heart conditions. However, these factors did not significantly change in either groups (data not shown).

Our study had several limitations. First, it was a single-center study with a small sample size, which may have concealed clinically significant differences between the education and control groups. We selected a country where the intake of salt is already high; this may serve as a bias. Therefore, further studies with a larger number of patients should be conducted on a wider level in various customs and traditions where the intake of sodium is not so common. Second, formula-derived estimates of 24-hour urinary excretion were used for the analyses. Spot urine samples collected during consultation alone may not reflect the actual urinary sodium and potassium intake levels. Third, our study involved only single-point measurements of 24hUNaV, 24hUKV, urinary Na/K ratio, SBP, DBP, and BMI after one year. It may be better to evaluate the transition of values in a shorter term if we investigate the effectiveness of our education program.

## Conclusions

This study is novel in the point that physicians may be able to reduce salt intake in outpatients with CKD by measuring estimated daily salt intake with a spot urine test and simple salt reduction education. However, this education did not reveal a significant reduction in BP as a result of salt reduction. Further studies with a larger number of patients are needed to confirm our findings.
